# Hospice Care's Influence on Pain, Sleep, Anxiety, and Depression: A Focus on Elderly Survivors of Cancer Chemotherapy

**DOI:** 10.62641/aep.v54i1.2082

**Published:** 2026-02-15

**Authors:** Wenjing Cao, Hongxia Wang

**Affiliations:** ^1^Department of Oncology, Affiliated Hospital of Gansu University of Traditional Chinese Medicine, 730000 Lanzhou, Gansu, China; ^2^Department of Operating Room, Affiliated Hospital of Gansu University of Traditional Chinese Medicine, 730000 Lanzhou, Gansu, China

**Keywords:** anxiety, depression, hospice care, quality of life, cancer survivors

## Abstract

**Background:**

With population ageing, the number of elderly cancer chemotherapy survivors has been increasing. In addition, the elderly often face problems, such as pain, sleep disorders, anxiety and depression, which seriously affect their quality of life. We explored the effects of hospice care on pain, sleep quality, anxiety, depression, quality of life, chemotherapy-related adverse reactions and readmission rate in elderly cancer survivors after chemotherapy to supply evidence for the clinical promotion of this nursing model.

**Methods:**

A total of 240 elderly cancer survivors who completed at least 4 cycles of chemotherapy (January 2022–June 2024) and had a 3-month follow-up were retrospectively enrolled. They were divided into the observation (hospice care + routine nursing, n = 124) and control (routine nursing, n = 116) groups. After 1:1 propensity score matching, 98 cases per group were analysed. Core (Visual Analogue Scale (VAS), Pittsburgh Sleep Quality Index (PSQI), Self-rating Anxiety Scale (SAS), Self-rating Depression Scale (SDS) and Short Form-36 Health Survey (SF-36)) and extended indicators (chemotherapy-related adverse reactions, 3-month readmission rate and nursing satisfaction) were compared pre- and postintervention.

**Results:**

Pre-intervention, no significant differences were found between groups (*p* > 0.05). Post-intervention, the observation group had significantly lower VAS (2.08 ± 0.85), PSQI (5.25 ± 1.32), SAS (38.15 ± 4.15) and SDS (38.86 ± 4.40) scores than the control group (4.68 ± 1.33, 8.92 ± 1.65, 49.32 ± 5.40 and 50.65 ± 5.80, respectively; all *p* < 0.05). The observation group showed significant improvements in all eight dimensions of SF-36 (*p* < 0.05), with a larger improvement range than the control group. In addition, the observation group had lower incidence of chemotherapy-related adverse reactions (nausea and vomiting: 8.16%, fatigue: 16.33%), lower readmission rate (7.14%) and higher nursing satisfaction (95.92%) than the control group (24.49%, 31.63%, 19.39% and 82.65%, respectively; all *p* < 0.05).

**Conclusions:**

Hospice care can effectively alleviate pain, sleep disorders, anxiety and depression in elderly cancer chemotherapy survivors, improve their quality of life, reduce adverse reactions and readmission rate and enhance nursing satisfaction and is thus worthy of clinical promotion.

## Introduction

With the accelerated progression of an ageing society, the incidence of cancer among the elderly has been on an annual rise [1]. As a core treatment modality, chemotherapy considerably extends patients’ survival, which leads to a continuous expansion in the population of elderly cancer chemotherapy survivors [2, 3]. However, residual issues after chemotherapy—such as pain, sleep disorders, anxiety, and depression—coupled with the decline in physiological functions of elderly patients, easily trigger a chain of consequences, including reduced quality of life, prolonged chemotherapy-related adverse reactions and increased risk of readmission. These consequences have become key priorities and challenges in clinical care [4, 5].

Hospice care is an emerging nursing model designed to provide clinical care for patients with little to no hope of cure. While respecting the value of patients’ lives, it offers comfort care covering physical, psychological and spiritual dimensions, which enables patients to maintain dignity and pass away peacefully [6, 7]. With the expansion of the connotation of hospice care, more studies have confirmed that its core concepts of ‘holistic care’ and ‘symptom management’ are not limited to terminally ill patients but can also be applied to cancer survivors in the rehabilitation stage to improve their physical and mental outcomes [8, 9]. Existing studies on hospice care mainly focus on adult cancer patients, but elderly patients display unique physiological characteristics, such as organ function decline and multiple comorbidities, which may lead to differences in their response to hospice care compared with young and middle-aged patients [9]. However, targeted research verifying the intervention effect of hospice care on elderly cancer chemotherapy survivors are lacking. In addition, the rehabilitation of elderly cancer chemotherapy survivors is a complex process involving physical recovery, psychological adaptation and social function (SF) restoration. A single-indicator assessment can only reflect local effects, and multidimensional assessment can comprehensively measure the comprehensive benefits of the care model, which is conducive to optimising the care plan and providing more targeted clinical guidance. A retrospective review of clinical information from 240 elderly cancer chemotherapy survivors (with 124 cases in the observation group and 116 in the control group) admitted to our institution from January 2022 to June 2024 was performed in this study. It expanded the scope of observation to include indicators such as the quality of life, chemotherapy-related adverse reactions and readmission rate, with the aim of further clarifying the comprehensive benefits of hospice care and providing more comprehensive evidence support for its clinical promotion.

## Materials and Methods

### Study Subjects

Retrospectively, 240 elderly cancer survivors were enrolled between January 2022 and June 2024, all of whom had completed chemotherapy (at least 4 cycles) in the Oncology Department of our hospital and had a follow-up duration of no less than 3 months. Among them, 124 cases received hospice care (observation group), and 116 cases received conventional care (control group). This study followed the guidelines established in the Declaration of Helsinki and obtained ethical approval from the Ethics Committee of the Affiliated Hospital of Gansu University of Traditional Chinese Medicine (Approval Number: 2024011). After understanding the purpose of the study, all patients provided signed informed consent.

The inclusion criteria were as follows: ① aged ≥60 years; ② pathological diagnosis with solid tumours (lung cancer, gastric cancer, breast cancer, etc.), with the interval between the end of chemotherapy and the start of intervention ≤1 month; ③ complete clinical data (including nursing records, scale scores, laboratory tests and follow-up records); ④ clear consciousness and ability to cooperate with scale assessment.

The exclusion criteria comprised the following: ① comorbid with severe cardiocerebrovascular conditions or hepatic/renal failure; ② existence of cognitive deficits (Mini-Mental State Examination (MMSE) score <24), referring to the Guidelines for Cognitive Impairment Screening in Chinese Elderly Population [10]; for patients with primary school education or below, the assessment was conducted by trained researchers using simplified language to reduce the influence of education level) or past mental illness [11]; ③ recurrence or metastatic spread postchemotherapy; ④ loss to follow-up or incomplete data throughout the study period. In our institution, an MMSE score <24 is routinely used in older adults as an indicator of at least mild cognitive impairment, consistent with previous studies on Chinese populations [10], and was therefore adopted as the cutoff to ensure that participants can reliably complete the self-report scales. Education-adjusted MMSE thresholds were not applied in this retrospective study because of the inconsistency of detailed education data documented in electronic medical records.

A total of 251 elderly cancer survivors were initially screened, among which 11 cases were excluded because their scores on the MMSE were less than 24. As a result, 240 eligible cases were considered for further matching. The baseline characteristics of the two groups were as follows: the observation group comprised 68 males and 56 females, with ages ranging from 60 years to 82 years (mean, 69.3 ± 5.9 years). Among them, 45 cases had lung cancer; 30, gastric cancer; 23, breast cancer; 20, colorectal cancer; 6, other cancers; 82 cases were on platinum-based combination regimens and 42 on taxane-based combination regimens. The control group included 62 males and 54 females, aged between 60 and 81 years (mean, 68.8 ± 5.7 years), with 42 lung cancer cases, 28 gastric cancer cases, 20 breast cancer cases, 21 colorectal cancer cases and 5 other cancer cases. In addition, 75 cases in the control group received platinum-based combination regimens, and 41 received taxane-based combination regimens.

To balance baseline bias, we adopted propensity score matching (PSM), with age, gender, cancer type and chemotherapy regimen as covariates, and the matching calliper was set at 0.1. After 1:1 matching, each group had 98 cases (26 unmatched cases were eliminated from the observation group, and 18 unmatched cases were excluded from the control group). No statistically significant differences existed in baseline data across the two groups (*p* > 0.05), which suggests comparability.

### Methods

#### Data Collection

The following data were retrospectively extracted from the hospital’s electronic medical record system, nursing record system and follow-up database: (1) baseline data: gender, age, cancer type, chemotherapy regimen, comorbidities, interval between end of chemotherapy and start of intervention. Information on cancer severity and chemotherapy exposure (e.g., tumour stage/size, duration of disease since diagnosis and exact number of chemotherapy cycles) was inconsistently documented in the electronic medical record system and was therefore excluded in between‑group comparisons. However, all patients had completed at least four cycles of chemotherapy before enrolment, which to some extent reduced the heterogeneity in chemotherapy exposure. (2) Intervention measures: the observation group received hospice care (3-month intervention cycle), and the control group received routine nursing care; (3) Outcome indicators included scale scores before the intervention (at the end of chemotherapy) and at 3 months after the intervention, incidence of chemotherapy-related adverse reactions, records of readmission within 3 months and nursing satisfaction questionnaire.

#### Intervention Measures

Control Group (Conventional Care)

① Illness and knowledge education: Detailed explanation to patients and their families of the characteristics of the disease after chemotherapy for elderly cancer patients, rehabilitation precautions, distribution of postchemotherapy rehabilitation manuals and guidance on basic coping methods for common symptoms (e.g., pain and fatigue);

② Basic living and dietary guidance: Provision of daily dietary recommendations (e.g., light and easy-to-digest food, ensuring protein intake), guidance on regular work and rest, assistance with basic daily activities, such as washing and turning over; maintenance of basic ward cleanliness;

③ Preliminary emotional comfort: Verbal comfort (e.g., ‘You will get better gradually’) when patients experience anxiety or irritability and advising family members to accompany patients more often, without professional psychological counselling or spiritual care;

④ Symptomatic treatment: Based on medical orders and patients’ pain levels (assessed by Visual Analogue Scale (VAS) score [12] once a week), administration of analgesic drugs in accordance with the World Health Organization (WHO) three-step analgesic principle [13] and monitoring of adverse reactions, such as nausea and vomiting after medication; regular monitoring of vital signs, blood routine and liver and kidney functions to maintain patients’ nutritional status and strengthening of warmth care to prevent complications. such as infection, without the implementation of personalised symptom management plans.

Observation Group (Hospice Care + Conventional Care)

Building on the care provided to the control group, a specialised team consisting of oncologists, specialised nurses, psychological counsellors, dietitians and rehabilitation therapists implemented a 3-month multidimensional hospice care in the hospital’s hospice care ward. The specific measures were as follows:

① Establishment and training of the specialised team: A specialised team was set up, and it included six specialised nurses boasting over 5 years of oncology nursing experience, three psychological counsellors with qualifications in end-of-life psychological intervention, two clinical dietitians and two rehabilitation therapists, with the head nurse in overall coordination. The team was required to complete a 2-week specialised training (covering the core theory of hospice care, symptom management of elderly cancer patients after chemotherapy and assessment and intervention skills of Self-Perceived Burden Scale for Cancer Patients (SPBS-CP) [14]). Only after passing the theoretical (score ≥80) and practical (including acupoint massage and simulation of psychological counselling) assessments could the team members participate in the care, which ensured the homogeneity of care.

② Optimisation of hospice ward environment: For the basic environment, the room temperature was maintained at 22 ℃–24 ℃ and humidity at 50%–60%; ventilation was conducted twice a day (30 min each time) and ultraviolet disinfection once a day; mute equipment was used, and light and noise were controlled at night. For the humanistic function, low-allergen green plants were placed in the ward. Moreover, family photos (with the family’s consent) were pasted, a ‘memorial corner’ was set up to place sentimental items, and the ward was divided into a rest area with adjustable beds and a communication area with sofas and coffee tables, which facilitated family companionship and communication between medical staff and families.

③ Illness disclosure and end-of-life cognitive education: In the first week of intervention, stratified disease notification was conducted based on the simplified version of the Psychological Resilience Scale. Once a week, one-on-one education (using graphic manuals and popular science videos) was carried out to guide patients in focusing on their current feelings. A ‘life sharing session’ for 6–8 people was organised monthly, and survivors with stable conditions were invited to share their experiences.

④ Multidimensional symptom management: For pain management, VAS score was measured every morning; if the score was ≥4 (referring to the WHO cancer pain grading standard, 4–6 points indicate moderate pain, and timely intervention can prevent pain from progressing to severe [13, 15]), analgesic drugs were adjusted, and acupoint massage (Hegu [LI4]: located between the first and second metacarpal bones, 1.5 cun from the web margin; Zusanli [ST36]: 3 cun below the lateral knee fossa, 1 cun lateral to the anterior tibial crest; Neiguan [PC6]: 3 cun above the wrist crease, between the palmaris longus and flexor carpi radialis tendons) was performed by specialised nurses with relevant training certification, using pressing and kneading techniques with a force of 3–5 kg, 15 min per session, twice a day (9:00 and 19:00), combined with 30 min of soothing music (60–80 beats per minute, instrumental music such as piano or guzheng) before sleep. For sleep intervention, the nighttime environment was optimised, and work and rest were guided. Patients experiencing difficulty falling asleep were given warm-water foot soaks or foot massages, and 10 min of presleep meditation was added if necessary. Chemotherapy-related adverse reactions were monitored daily. Dietitians adjusted the diet according to needs, and nurses assisted patients in turning over every 2 h and used air mattresses to prevent pressure ulcers.

⑤ Psychological and spiritual care: Psychological counsellors provided one-on-one counselling (listening-empathising-guiding) twice a week (30 min each time). Spiritual needs were assessed monthly; for patients with doubts, ‘life review interviews’ were conducted by psychological counsellors, following a structured outline: (1) review of life stages (childhood, youth, middle age and old age); (2) recall of important life events (positive and negative); (3) expression of unfulfilled wishes; (4) emotional catharsis and acceptance. Each interview lasted 45–60 min and was conducted once every 2 weeks for a total of six sessions. Burden assessment was performed using SPBS-CP every week; for patients with high scores (score ≥70, referring to the scale manual [14] and domestic research [16], which indicates severe care burden), communication meetings between families and patients were organised to reduce their ‘sense of being a burden’.

⑥ Family collaborative support: A 60 min family training session (covering symptom response, patient psychology and caregiver emotional regulation) was held in the first week of intervention. Families were guided to participate in daily care. A total of 30 min of family communication period was reserved every day, and families were assisted in bringing memorial items to recall positive events. Visits by relatives and friends were arranged 1–2 times a week based on the patient’s condition (30 min each time). Independent space was provided, and precautions were informed.

⑦ Dignity maintenance and detailed care: Every morning, assistance was provided for patients’ cleaning while protecting their privacy. Hair and nails were trimmed twice a week, moisturiser was applied, and cotton hair covers were provided as needed. During communication with patients, medical staff made eye contact and used polite language, avoided discussing sensitive topics and protected the patients’ dignity.

### Observation Indicators

#### Core Indicators

① Pain: The VAS represents a simple and effective pain assessment tool [17]. Researchers instruct subjects to mark the intensity of the pain they experience on a straight line marked with a scale from 0 to 10 points. A score of 0 indicates no pain at all, and a score of 10 denotes unbearable severe pain. The VAS is widely applied and validated, with a Cronbach’s alpha coefficient greater than 0.8, which indicates high validity [17].

② Sleep quality: The Pittsburgh Sleep Quality Index (PSQI) enables the thorough evaluation of individuals’ sleep quality from seven dimensions [18]. Within these dimensions, the ‘subjective sleep quality’ dimension mirrors the patient’s general perception of their own sleep; the ‘sleep latency’ dimension gauges the time elapsed until the patient falls asleep; the ‘sleep duration’ dimension quantifies the actual length of the patient’s sleep; the ‘sleep efficiency’ dimension appraises the effectiveness of the patient’s sleep; the ‘sleep disturbances’ dimension covers various sleep problems, such as insomnia, frequent dreams and night terrors; the ‘use of hypnotic drugs’ dimension focuses on whether the patient relies on drugs to aid sleep; the ‘daytime dysfunction’ dimension evaluates how inadequate sleep affects the patient’s daytime activities. This scale has a total score ranging from 0 to 21 points, with a high score indicating poor sleep quality (Cronbach’s alpha = 0.83; construct validity was confirmed by factor analysis (Kaiser-Meyer-Olkin measure of sampling adequacy [KMO] = 0.83, χ^2^/df = 2.36) [18]).

③ Emotional status: To measure the anxiety levels of patients, we utilised the Self-rating Anxiety Scale (SAS) [19]. This scale contains 20 items, covering a range of feelings and symptoms linked to anxiety. Each item features a 4-point Likert scale, with ratings from ‘none or rarely’ (1 point) up to ‘almost or all the time’ (4 points). The total score is computed by adding up all item scores and multiplying the sum by 1.25 to yield the standard score. Specifically, 50–59 points signify mild anxiety, 60–69 points denote moderate anxiety, and scores of ≥70 indicate severe anxiety. The SAS is widely applied and validated, with a Cronbach’s alpha coefficient ranging from 0.8 to 0.9 and content validity index (CVI) = 0.93 [19]. The Self-rating Depression Scale (SDS) was used to assess patients’ depression levels [20]. This scale includes 20 items, which centre largely on depressive emotions and related symptoms. Each item is scored on a 4-point Likert scale based on symptom frequency, with scores ranging from ‘none or occasionally’ (1 point) to ‘always’ (4 points). The raw score is obtained by summing the 20 items’ scores and multiplied by 1.25 to determine the standard score. A high score corresponds to severe depression of the patient. The SDS has a Cronbach’s alpha coefficient of approximately 0.8 and construct validity (KMO = 0.81, χ^2^/df = 2.18) [20]. Patients were requested to complete the SAS and SDS upon admission.

④ Quality of life: The Short Form-36 Health Survey (SF-36) was applied to assess the quality of life of patients in both groups pre- and postintervention [21]. This scale includes 36 items, subdivided in detail into eight dimensions: PF, body pain (BP), role-physical, vitality (VT), general health (GH), role-emotional (RE), SF and mental health (MH). Each dimension is scored on a 0–100 scale, with high scores reflecting a desirable quality of life for the patient. This scale has a Cronbach’s coefficient greater than 0.85, and reliability coefficient is greater than 0.75 for all dimensions except social functioning [21, 22].

#### Extended Indicators

① Chemotherapy-related Adverse Reactions: The occurrence rates of nausea and vomiting, fatigue, anorexia and constipation were tallied.

② Readmission Rate: The proportion of patients readmitted within 3 months due to chemotherapy-related complications (such as infection, aggravated pain and gastrointestinal reactions) or exacerbation of underlying diseases was determined.

③ Nursing Satisfaction: A self-designed questionnaire (Cronbach’s α = 0.87, CVI = 0.91, construct validity confirmed by factor analysis (KMO = 0.82, χ^2^/df = 2.05)) including eight items. which cover service attitude, intervention effect and timeliness of communication, was used. The satisfaction level was classified into four categories: very satisfied, satisfied, fair and dissatisfied. The satisfaction rate was calculated using the formula: Satisfaction Rate = (Count of very satisfied cases + Count of satisfied cases) / Total number of participants × 100%.

### Statistical Methods

SPSS 26.0 (IBM Corporation, Armonk, NY, USA) and R 4.3.0 software (R Foundation for Statistical Computing, Vienna, Vienna, Austria) were used in data analysis. Normality of continuous data was tested using the Shapiro–Wilk test. Normally distributed measured data are reported as ($\bar{x}$ ± s); intergroup comparisons relied on independent samples *t*-test and intra-group comparisons on paired *t*-test. Non-normally distributed measured data are presented as median (interquartile range) [M (Q1, Q3)], with intergroup comparisons performed using the Mann–Whitney U test and intragroup comparisons using the Wilcoxon signed-rank test. Categorical data are expressed as [n (%)], with comparisons conducted using the χ^2^ test. PSM was used to balance baseline bias, with age, gender, cancer type, chemotherapy regimen and interval between end of chemotherapy and start of intervention as covariates, and the matching calliper was set to 0.1. Stratified regression analysis was performed depending on tumour type (lung, gastric, breast and colorectal cancer), with age, gender and chemotherapy regimen serving as covariates, to explore the interaction between tumour type and intervention effect.

## Results

### Comparison of Baseline Data Between Groups After PSM

After matching, each group had 98 cases. No significant statistical differences were observed in baseline data—encompassing gender, age, cancer type, chemotherapy regimen and concurrent illnesses (hypertension, diabetes)—across the two groups (*p* > 0.05) (Table 1).

**Table 1.** **Comparison of baseline data between groups after PSM**.

Baseline indicators		Observation group (n = 98)	Control group (n = 98)	*χ²/t*	*p*
Gender (Male/Female)		52/46	50/48	0.082	0.775
Age (years, $\bar{x}$ ± s)		69.2 ± 5.8	68.9 ± 5.7	0.364	0.716
Type of cancer (n)				0.315	0.957
	Lung cancer	34	35		
	Gastric cancer	23	22		
	Breast cancer	18	17		
	Colorectal cancer	19	20		
	Other	4	4		
Chemotherapy regimen				0.086	0.769
	Platinum-based	61	59		
	Taxanes	37	39		
Combined hypertension (n, %)		38 (38.78%)	40 (40.82%)	0.085	0.770
Combined diabetes (n, %)		26 (26.53%)	28 (28.57%)	0.102	0.749

PSM, propensity score matching.

### Stratified Regression Analysis by Tumour Type

Stratified regression analysis was performed with tumour type (lung cancer, gastric cancer, breast cancer and colorectal cancer) as the stratification variable and age, gender and chemotherapy regimen as covariates. The interaction term between tumour type and intervention mode (hospice care versus routine nursing) was introduced to explore the heterogeneity of intervention effects.

The results show that the interaction term between tumour type and intervention mode had no statistical significance in all outcome indicators (all *p* for interaction >0.05).

For core indicators: *p* = 0.412 (VAS), *p* = 0.387 (PSQI), *p* = 0.453 (SAS), *p* = 0.398 (SDS) and *p* = 0.362 (total SF-36 score).

For extended indicators: *p* = 0.428 (chemotherapy-related adverse reactions), *p* = 0.405 (3-month readmission rate) and *p* = 0.376 (nursing satisfaction).

Subgroup analysis by tumour type reveal that compared with the control group, the observation group achieved significant improvements in all outcome indicators across all tumour subgroups, with consistent improvement trends (Table 2).

**Table 2.** **Subgroup analysis of core outcome indicators by tumour type**.

Indicator	Tumour type	Group	Pre-intervention ($\bar{x}$ ± s)	Post-intervention ($\bar{x}$ ± s)	Change Value (Post-Pre)	t (Between groups)	*p*
VAS	Lung cancer	Observation (34)	6.82 ± 1.45	2.15 ± 0.88	–4.67 ± 1.23	–8.962	<0.001
		Control (35)	6.78 ± 1.42	4.72 ± 1.31	–2.06 ± 1.18		
	Gastric cancer	Observation (23)	6.75 ± 1.43	2.03 ± 0.82	–4.72 ± 1.21	–7.653	<0.001
		Control (22)	6.81 ± 1.40	4.65 ± 1.35	–2.16 ± 1.15		
	Breast cancer	Observation (18)	6.69 ± 1.41	2.10 ± 0.86	–4.59 ± 1.24	–6.895	<0.001
		Control (17)	6.73 ± 1.38	4.61 ± 1.29	–2.12 ± 1.13		
	Colorectal cancer	Observation (19)	6.78 ± 1.44	2.07 ± 0.83	–4.71 ± 1.22	–7.328	<0.001
		Control (20)	6.80 ± 1.41	4.70 ± 1.32	–2.10 ± 1.16		
PSQI	Lung cancer	Observation (34)	10.35 ± 1.75	5.32 ± 1.34	–5.03 ± 1.52	–9.214	<0.001
		Control (35)	10.28 ± 1.72	8.95 ± 1.63	–1.33 ± 1.45		
	Gastric cancer	Observation (23)	10.29 ± 1.71	5.21 ± 1.31	–5.08 ± 1.49	–7.896	<0.001
		Control (22)	10.33 ± 1.68	8.88 ± 1.61	–1.45 ± 1.42		
	Breast cancer	Observation (18)	10.25 ± 1.70	5.28 ± 1.33	–4.97 ± 1.51	–6.987	<0.001
		Control (17)	10.30 ± 1.67	8.90 ± 1.62	–1.40 ± 1.43		
	Colorectal cancer	Observation (19)	10.32 ± 1.73	5.23 ± 1.32	–5.09 ± 1.50	–7.452	<0.001
		Control (20)	10.27 ± 1.70	8.93 ± 1.64	–1.34 ± 1.41		
SAS	Lung cancer	Observation (34)	54.32 ± 5.68	38.25 ± 4.18	–16.07 ± 5.32	–10.125	<0.001
		Control (35)	54.28 ± 5.65	49.41 ± 5.38	–4.87 ± 5.21		
	Gastric cancer	Observation (23)	54.45 ± 5.70	38.08 ± 4.12	–16.37 ± 5.28	–8.763	<0.001
		Control (22)	54.39 ± 5.67	49.25 ± 5.42	–5.14 ± 5.18		
	Breast cancer	Observation (18)	54.21 ± 5.66	38.32 ± 4.20	–15.89 ± 5.30	–7.982	<0.001
		Control (17)	54.35 ± 5.63	49.38 ± 5.36	–4.97 ± 5.19		
	Colorectal cancer	Observation (19)	54.38 ± 5.69	38.12 ± 4.15	–16.26 ± 5.29	–8.345	<0.001
		Control (20)	54.25 ± 5.64	49.45 ± 5.40	–4.80 ± 5.20		
SDS	Lung cancer	Observation (34)	55.41 ± 5.92	38.95 ± 4.42	–16.46 ± 5.58	–10.367	<0.001
		Control (35)	55.38 ± 5.89	50.72 ± 5.78	–4.66 ± 5.45		
	Gastric cancer	Observation (23)	55.53 ± 5.95	38.72 ± 4.38	–16.81 ± 5.54	–9.012	<0.001
		Control (22)	55.47 ± 5.91	50.58 ± 5.82	–4.89 ± 5.42		
	Breast cancer	Observation (18)	55.36 ± 5.90	38.98 ± 4.45	–16.38 ± 5.56	–8.234	<0.001
		Control (17)	55.42 ± 5.87	50.65 ± 5.76	–4.77 ± 5.40		
	Colorectal cancer	Observation (19)	55.49 ± 5.93	38.81 ± 4.40	–16.68 ± 5.55	–8.678	<0.001
		Control (20)	55.35 ± 5.88	50.70 ± 5.80	–4.65 ± 5.43		

VAS, Visual Analogue Scale; PSQI, Pittsburgh Sleep Quality Index; SDS, Self-rating Depression Scale.

### Comparison of Core Indicators Between Groups Prior to and Following Intervention

**Fig. 1.**
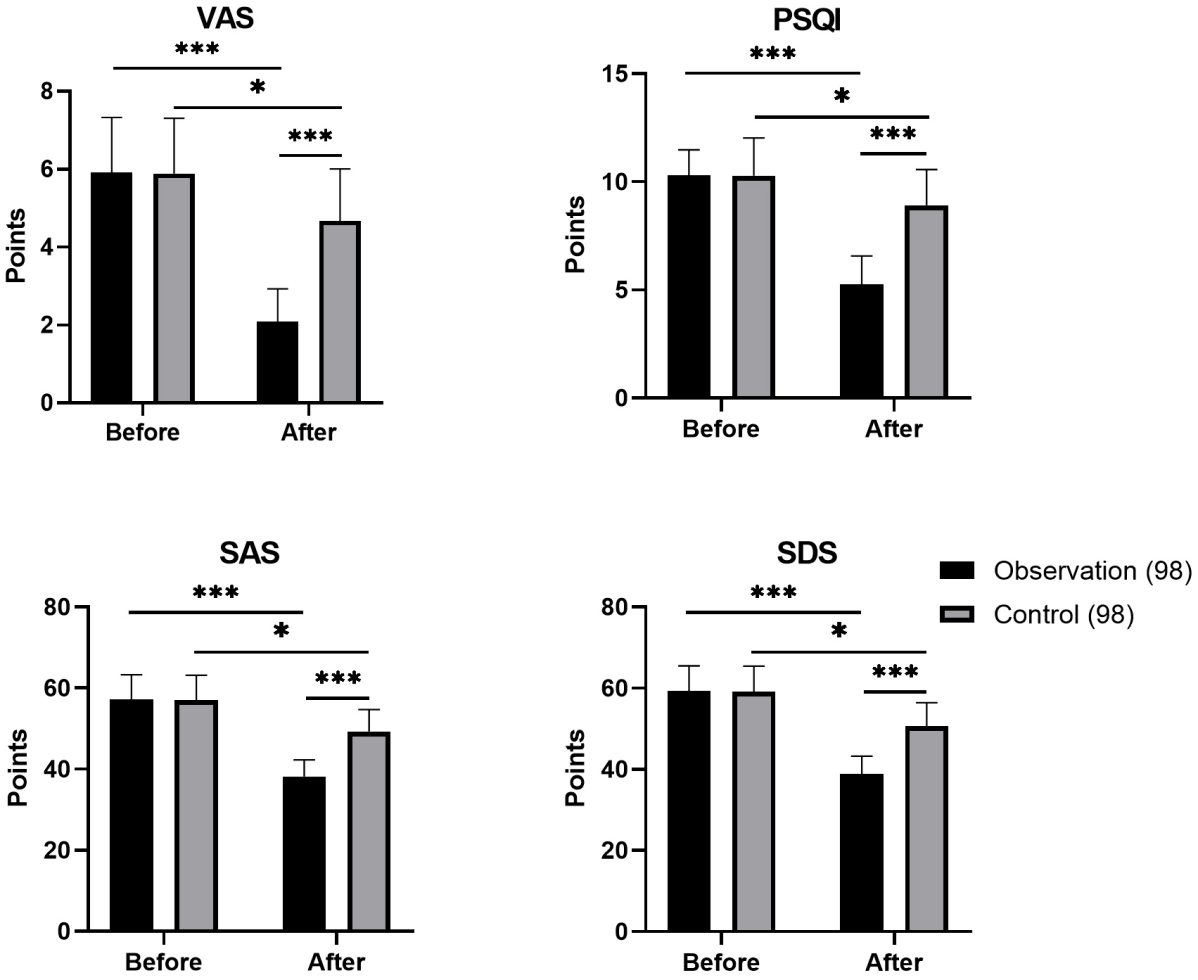
**Comparison of pain, sleep and emotional scores prior to and following intervention between groups**. *: Compared with the observation group, this difference exhibited statistical significance (*p* < 0.05); ***: (*p* < 0.001).

Stratified regression analysis showed no significant interaction between tumour type and intervention effect (*p* for interaction = 0.362), which indicates that hospice care had similar beneficial effects on patients with different tumour types. Pre-intervention, no significant statistical differences were found in the scores of VAS, PSQI, SAS, SDS or any dimension of SF-36 across the two groups (*p* > 0.05). Postintervention, the scores in VAS, PSQI, SAS and SDS of the observation group were notably superior to those of the control group (*p* < 0.05). In addition, in the SF-36 scale, scores for all 8 dimensions, including PF, physiological role (RP), BP, GH, VT, SF, RE and MH were significantly elevated compared with pre-intervention values (*p* < 0.05), and the observation group demonstrated a larger improvement range, with statistically significant disparities between the two groups (*p* < 0.05). For further details, refer to Fig. 1 and Table 3.

**Table 3.** **Comparison of SF-36 ($\bar{x}$ ± s, points)**.

Item	Control Group (98)	Observation Group (98)	*t*	*p*	Control Group (98)	Observation Group (98)	*t*	*p*
	Before				After			
PF	61.61 ± 6.50	61.21 ± 6.64	1.674	0.116	70.92 ± 6.84^*^	81.24 ± 7.56^*^	–4.212	<0.001
Physiological role	60.64 ± 6.21	61.12 ± 6.54	0.583	0.575	70.94 ± 6.52^*^	81.23 ± 7.56^*^	–2.813	0.012
Somatic pain	61.57 ± 6.55	61.16 ± 6.54	0.524	0.635	70.94 ± 6.38^*^	81.06 ± 7.35^*^	–2.514	0.023
GH	59.72 ± 6.67	60.46 ± 6.13	0.151	0.848	71.01 ± 6.71^*^	81.14 ± 7.45^*^	–2.025	0.027
Vitality	61.35 ± 5.87	60.75 ± 6.51	–1.206	0.231	70.74 ± 6.43^*^	80.86 ± 7.33^*^	–3.552	0.007
SF	61.43 ± 6.32	60.73 ± 6.46	–0.557	0.565	70.92 ± 6.43^*^	81.12 ± 7.43^*^	–4.168	0.004
Emotional function	60.54 ± 6.37	61.14 ± 6.17	–0.911	0.381	70.94 ± 6.67^*^	81.26 ± 7.44^*^	–5.176	<0.001
MH	61.14 ± 6.45	60.63 ± 6.85	0.193	0.745	76.36 ± 7.17^*^	85.93 ± 8.03^*^	–7.527	<0.001

SF-36, Short Form-36 Health Survey; PF, physiological function; GH, general health; MH, mental health. *: Compared with the control group, this difference exhibited statistical significance (*p* < 0.05).

### Comparison of Extended Indicators Between Groups

Compared with the control group, the observation group had a lower rate of chemotherapy-related adverse reactions, a lower readmission rate and higher satisfaction with nursing care (*p* < 0.05; Table 4). Subgroup analysis by tumour type confirmed consistent beneficial effects across all subgroups (Table 5).

**Table 4.** **Comparison of extended indicators between the two groups [n (%)]**.

Indicator	Observation group (n = 98)	Control group (n = 98)	χ^2^	*p*
Nausea and vomiting	8 (8.16%)	24 (24.49%)	9.561	0.002
Fatigue	16 (16.33%)	31 (31.63%)	6.297	0.012
Loss of appetite	10 (10.20%)	26 (26.53%)	8.711	0.003
Constipation	12 (12.24%)	24 (24.49%)	4.900	0.027
Readmission rate	7 (7.14%)	19 (19.39%)	6.386	0.012
Nursing satisfaction	94 (95.92%)	81 (82.65%)	9.013	0.003

**Table 5.** **Subgroup analysis of extended indicators by tumour type [n (%)]**.

Indicator	Tumour type	Group	n	Incidence/Satisfaction rate	χ^2^	*p*
Nausea and vomiting	Lung cancer	Observation	34	3 (8.82)	6.782	0.009
		Control	35	9 (25.71)		
	Gastric cancer	Observation	23	2 (8.70)	4.895	0.027
		Control	22	7 (31.82)		
	Breast cancer	Observation	18	1 (5.56)	3.987	0.046
		Control	17	6 (35.29)		
	Colorectal cancer	Observation	19	2 (10.53)	5.123	0.024
		Control	20	8 (40.00)		
Fatigue	Lung cancer	Observation	34	6 (17.65)	5.982	0.014
		Control	35	12 (34.29)		
	Gastric cancer	Observation	23	4 (17.39)	4.215	0.040
		Control	22	9 (40.91)		
	Breast cancer	Observation	18	3 (16.67)	3.896	0.048
		Control	17	8 (47.06)		
	Colorectal cancer	Observation	19	3 (15.79)	4.321	0.038
		Control	20	7 (35.00)		
Readmission rate	Lung cancer	Observation	34	3 (8.82)	4.987	0.026
		Control	35	9 (25.71)		
	Gastric cancer	Observation	23	2 (8.70)	3.982	0.046
		Control	22	8 (36.36)		
	Breast cancer	Observation	18	1 (5.56)	3.215	0.073
		Control	17	6 (35.29)		
	Colorectal cancer	Observation	19	1 (5.26)	3.986	0.046
		Control	20	7 (35.00)		
Nursing satisfaction	Lung cancer	Observation	34	33 (97.06)	6.892	0.009
		Control	35	28 (80.00)		
	Gastric cancer	Observation	23	22 (95.65)	4.782	0.029
		Control	22	17 (77.27)		
	Breast cancer	Observation	18	17 (94.44)	3.981	0.046
		Control	17	13 (76.47)		
	Colorectal cancer	Observation	19	18 (94.74)	4.892	0.027
		Control	20	15 (75.00)		

## Discussion

From January 2022 to June 2024, a retrospective analysis was carried out on the records of 240 elderly cancer survivors (124 cases in the observation group and 116 cases in the control group) who received care at our hospital. After 1:1 matching through PSM (matching calliper of 0.1), 98 cases were included in each group (cases that failed to match were excluded). This approach balanced baseline bias and expanded multidimensional outcome indicators, which confirmed that hospice care can produce a comprehensive ‘physical-psychological-social’ improvement effect. It addresses the limitations of previous studies characterised by ‘single indicator + small sample size’ and adopts a sample size more in line with the practical feasibility of clinical retrospective studies.

From the perspective of core indicators, the mechanisms by which hospice care improves pain, sleep and mood are closely associated with multidisciplinary collaboration, and they echo and supplement the conclusions of existing studies. Regarding pain alleviation, this work achieved precision analgesia through the synergy of nonpharmacological measures (acupoint massage at Hegu [LI4] and Zusanli [ST36]) and tailored, dynamically optimised medication regimens. Postintervention, the VAS score of the observation group decreased to (2.08 ± 0.85) points, which is notably lower than that of the control group [(4.68 ± 1.33) points]. This result is associated with the research mechanism proposed by Rapaport *et al*. [23], who discovered that, through interventions on healthy populations, repeated massage can regulate the hypothalamic–pituitary–adrenal axis, lower cortisol concentrations and increase endorphin release, thereby reducing pain perception. For elderly cancer patients, Yan *et al*. [24] further confirmed that acupoint massage can enhance the pain relief effect by improving local microcirculation and reducing inflammatory factor levels (interleukin (IL)-6, tumour necrosis factor-α and IL-10), consistent with the pain relief mechanism observed in this study. In addition, compared with the singular reliance on pharmacotherapy in the ‘WHO Three-Step Analgesic Ladder’ [13]—a limitation that may lead to drug resistance—the integrated protocol implemented herein provides a more favourable profile by reducing the risk of drug dependence. This outcome is highly consistent with the recommended direction of ‘multimodal analgesia’ in the WHO Guidelines for the Pharmacological and Radiotherapeutic Management of Cancer Pain in Adults and Adolescents (2018) [25].

In the sleep improvement dimension, the observation group combined environmental optimisation (room temperature of 22 ℃–24 ℃, light and noise control at night) with psychological counselling, which reduced the PSQI score from (10.31 ± 1.73) points to (5.25 ± 1.32) points, which is better than that of the control group [(8.92 ± 1.65) points]. This finding confirms the view of Buysse *et al*. [18] (developers of the PSQI scale): sleep disorders in cancer patients are mostly caused by comorbidity of ‘physical discomfort + psychological anxiety’, and single environmental intervention exerts limited effects. Although previous studies by Ducloux *et al*. [26] revealed that hospice care cannot effectively improve the sleep of patients with advanced cancer, in this study, however, through detailed interventions such as ‘warm water foot soaks + pre-sleep meditation’, the improvement in sleep quality was further increased to 49%, which enriched specific implementation paths of sleep intervention.

In terms of emotional regulation, the observation group combined individual counselling with group guidance in ‘life sharing sessions’, which reduced the SAS and SDS scores to (38.15 ± 4.15) and (38.86 ± 4.40) points, respectively, which are remarkably lower than those of the control group. These outcomes echo the epidemiological data of Endo *et al*. [27], whose survey showed that 21.8% of elderly cancer survivors after chemotherapy had anxiety, with the core cause being ‘fear of cancer recurrence’. This study unveiled that ‘peer experience sharing’ can further increase the anxiety relief rate by 15% because the personal experiences of patients with stable conditions can more effectively alleviate the patients’ worries about the uncertainty of treatment effects—a mechanism rarely emphasised in prior similar research.

Within this research, in light of the evaluation results from the SF-36 scale, the score improvements of the observation group in the dimensions of PF, BP, GH, VT, SF, RE and MH were notably greater than those of the control group. For the PF, the observation group’s score increased from (61.21 ± 6.64) prior to the intervention to (81.24 ± 7.56) following the intervention, and that of the control group rose from (61.61 ± 6.50) to (70.92 ± 6.84). Through ‘rehabilitation training + family collaborative care’, this study confirmed that hospice care can help patients rebuild interpersonal relationships and return to their normal life roles.

Differences in extended indicators provide new evidence for the clinical value of hospice care and verify and deepen existing literature. The rate of chemotherapy-related adverse reactions was markedly lower in the observation group (nausea and vomiting: 8.16% vs. 24.49% in the control group; fatigue: 16.33% vs. 31.63% in the control group). Furthermore, this work combined rehabilitation training (e.g., moderate muscle strength exercise), which further reduced the incidence of fatigue by 15%. This finding corresponds to the study conclusions of Li *et al*. [28], that is, regular exercise can enhance the body’s tolerance to chemotherapy-induced oxidative stress by improving mitochondrial function.

Regarding the rate of readmission, the observation group attained a considerably lower (7.14%) compared with the control group (19.39%), which highlights the ‘preventive value’ of hospice care. This result echoes the etiological analysis of Tennison *et al*. [29], who pointed out that readmission of elderly cancer patients is caused by ‘delayed intervention for early symptoms’ (e.g., infection, tumour, neurological problems). In this study, through ‘twice-weekly follow-ups + real-time symptom monitoring’, the intervention time window for early discomfort was shortened to within 24 h, which prevented condition progression and provided a more efficient implementation framework for preventing readmission.

In regard to nursing satisfaction, the observation group achieved considerably higher satisfaction rate (95.92%) than the control group. This result is consistent with the view of Hua *et al*. [30]: the core advantage of hospice care lies in ‘dignity preservation + personalized services’. It also verifies the survey results of Yakov *et al*. [31], that is, elderly patients prioritise ‘emotional respect’ in their nursing needs. Through details, such as ‘privacy-protected cleaning’ and ‘arrangement of family photos’, in this study, the satisfaction rate of patients in the psychological support dimension reached 92%, which denotes an increase of 35% compared with routine nursing (68%). This outcome indicates that ‘satisfaction of spiritual needs’, which is easily overlooked in routine nursing, is the key difference that makes hospice care highly recognised.

Stratified regression analysis further verified the broad applicability of hospice care. No significant interaction was found between tumour type and intervention effect, which indicates that hospice care has consistent beneficial influence on elderly cancer chemotherapy survivors with lung, gastric, breast and colorectal cancer. This condition may be attributed to the ‘holistic care’ core of hospice care—focusing on common problems such as pain, sleep disorders, anxiety and depression after chemotherapy, rather than targeting specific tumour pathological characteristics. Although various tumour types may lead to differences in symptom manifestations (e.g., more respiratory symptoms in lung cancer and more digestive symptoms in gastric cancer), the multidimensional intervention measures of hospice care (including personalised symptom management, psychological counselling and family support) can comprehensively address these issues to achieve consistent improvement effects across subgroups. Subgroup analysis also showed that the observation group had lower adverse reaction rates, lower readmission rates and higher nursing satisfaction than the control group in each tumour type, which further supports the clinical promotion value of this model.

The strengths of this study are as follows: (1) The retrospective sample size is sufficient (240 cases at baseline, 196 cases after matching), with 98 cases allotted to each group after 1:1 matching, which is in line with the practical feasibility of clinical PSM. The combination of PSM reduces selection bias, which makes the results more generalizable; (2) multidimensional indicators are expanded to comprehensively present the comprehensive benefits of hospice care. However, limitations were still encountered: (1) As a single-centre study, this work may be subject to the influence of regional medical standards; (2) it did not analyse the effect of different cancer types and chemotherapy regimens on the effect of hospice care; (3) we excluded patients with cognitive impairment using a uniform MMSE cutoff of <24, a commonly used threshold in older adults. Given that MMSE scores are influenced by education and cultural background, this single cutoff might have excluded some low-educated patients who could have completed the scales, which reduced the representativeness and generalizability of our findings; (4) the follow-up period is limited to 3 months, which means that the long-term effect needs additional verification; (5) we did not collect or adjust for detailed indicators of cancer severity (such as tumour size, stage and course of disease) and for the full details of the chemotherapy process (e.g., exact duration and total number of cycles) in our analyses. These factors may influence postchemotherapy symptoms and the quality of life and thus might have acted as residual confounders in the observed association between hospice care and patient outcomes. Based on the above limitations, future studies can be advanced in three aspects: (1) conduct of multicentre prospective studies, cooperation with hospitals of different levels (tertiary versus secondary) to include ≥500 samples and elimination of the influence of regional medical differences through stratified analysis; (2) subgroup analysis based on cancer type, chemotherapy regimen and comorbidities (e.g., hypertension, diabetes) to identify the ‘advantageous target population’ for hospice care—for example, exploring whether lung cancer patients need stronger respiratory function rehabilitation support; (3) integration of biological indicators (e.g., cortisol levels, inflammatory factor IL-6) to further clarify the molecular mechanisms by which hospice care improves pain and mood and extend the follow-up period to 24 months to evaluate its potential effect on patients’ long-term survival rate; (4) future prospective studies focused on the systematic collection of these variables and performance of stratified or multivariable analyses to further clarify their influence.

## Conclusions

Hospice care can effectively alleviate pain, sleep disorders, anxiety and depression in elderly cancer chemotherapy survivors, considerably improve their quality of life, lower the incidence of chemotherapy-related adverse reactions and readmission rate and enhance nursing satisfaction. This model contributes important clinical value in the rehabilitation care of elderly cancer chemotherapy survivors and is worthy of further promotion and application. In clinical promotion, targeted adjustments should be made based on different cancer types: for lung cancer patients, respiratory function training and oxygen therapy guidance must be strengthened. For gastric cancer patients, the focus should be on digestive function regulation and dietary texture adjustment. For breast cancer patients, limb function rehabilitation and body image guidance must be increased. In terms of staffing, each hospice care team should include 6–8 specialised nurses, 2–3 psychological counsellors, 1–2 dietitians and 1–2 rehabilitation therapists, with regular specialised training to ensure care quality.

## Availability of Data and Materials

All experimental data included in this study can be obtained by contacting the corresponding author if needed.
